# Up-regulated autophagy: as a protective factor in adipose tissue of WOKW rats with metabolic syndrome

**DOI:** 10.1186/s13098-018-0317-6

**Published:** 2018-03-02

**Authors:** J. Kosacka, M. Nowicki, S. Paeschke, P. Baum, M. Blüher, N. Klöting

**Affiliations:** 1 Department of Neurology, University of Leipzig, Liebigstraße 20, 04103 Leipzig, Germany; 2 Department of Medicine, University of Leipzig, Liebigstraße 21, 04103 Leipzig, Germany; 3 Institute of Anatomy, University of Leipzig, Oststraße 25, 04317 Leipzig, Germany; 4 Integrated Research and Treatment Center (IFB) Adiposity Diseases, Liebigstraße 19-21, 04103 Leipzig, Germany

**Keywords:** Autophagy, Adipose tissue, LY294002 inhibitor, Metabolic syndrome, WOKW rats

## Abstract

**Background:**

Wistar Ottawa Karlsburg W (RT1u) rats (WOKW) are a model of the metabolic syndrome (MetS). Adipose tissue (AT) and peripheral nerves of WOKW rats exhibit up-regulated autophagy and inflammation corresponding with decreased apoptosis rate. The aim of this study was to characterize AT in WOKW rats in relation to autophagic activity.

**Methods:**

mRNA and protein expression of adiponectin, pro-inflammatory and pro-apoptotic markers including MCP1, TNFα, cleaved caspase-3 and RNF157, a new candidate gene regulated through autophagy, were analyzed in adipocytes isolated from visceral and subcutaneous AT of 5-month old WOKW rats with MetS and LEW.1W controls in response to pharmacological inhibition of autophagy. Immunohistochemistry was performed to detect adiponectin and RNF157 protein in cultured adipocytes.

**Results:**

Inhibition of autophagy by LY294002 was associated with a fourfold up-regulation of adiponectin expression and a decrease of RNF157 protein and pro-inflammatory markers—MCP-1 and TNFα predominantly in visceral adipocytes of obese WOKW rats compared to LEW.1W rats. Moreover, inhibition of autophagic activity correlates with an activation of cleaved caspase-3 apoptotic signaling pathway.

**Conclusions:**

Up-regulated autophagy in obese WOKW rats contributes to the regulation of visceral AT function and involves an altered balance between pro-inflammatory and protective adipokine expression. Our data suggest that activation of AT autophagy protects against adipocyte apoptosis at least under conditions of obesity related MetS in WOKW rats.

**Electronic supplementary material:**

The online version of this article (10.1186/s13098-018-0317-6) contains supplementary material, which is available to authorized users.

## Background

The metabolic syndrome (MetS) characterized by obesity, hypertension, hyperinsulinemia, dyslipidemia and impaired glucose metabolism represents a risk factor for cardiovascular diseases and type 2 diabetes. In addition, a pro-inflammatory state may contribute to diabetic peripheral neuropathy, nephropathy, retinopathy and macrovascular complications [1–6].

Inflammation of adipose tissue (AT)—a symptom of AT dysfunction both in humans and model organisms which develops with weight gain and diabetes—is associated with AT autophagic activity [7–9]. Autophagy is an essential, lysosomal degradation process, which is implicated in cellular homeostasis through break down and recycling of aggregated proteins and damaged organelles [10]. However, activated autophagy has been generally considered as a cell-protection mechanism, which promotes cell survival; the excessive activation of autophagy may lead to autophagic cell death [11].

Autophagy has been shown to prevent or induce inflammatory responses and to regulate of cytokine production in AT of obesity in humans and animal models [12–14]. The molecular mechanisms underlying the interaction between altered immune-response and autophagy regulation in obesity are still poor investigated.

Recently, we have shown that enhanced autophagy characterized by up-regulated expression of autophagy genes in AT and peripheral nerves occurs in obese Wistar Ottawa Karlsburg W (RT1u) (WOKW) rats, a model for MetS [15, 16]. WOKW rats with the MHC RT1u haplotype develop a polygenetically inherited metabolic syndrome with obesity, hypertension, dyslipidemia and hyperinsulinemia, closely reflecting the human MetS [17–19]. Up-regulation of autophagy in WOKW rats corresponded with macrophage infiltration in the sciatic nerves and AT without signs of neuropathy or diabetes. Moreover, activated autophagy seems to protect from caspase-3 mediated apoptosis in AT and nerves of WOKW rats [15, 16]. There is strong evidence for a causal relationship between autophagy and AT function in obesity.

Here, we tested the hypothesis that autophagic activity contributes to the regulation of protective, inflammatory and apoptotic factors in adipocytes isolated from visceral and subcutaneous AT of WOKW rats. We provide data suggesting that AT autophagy represents a key mechanism protecting against the development of obesity-related metabolic comorbidities.

## Methods

### Animals

Five-month old WOKW (n = 12) and LEW.1W (n = 12) male rats were bred and kept under standard conditions maintained at 21 ± 1 °C on a 12:12 h light/dark cycle (5 a.m./5 p.m.) at the Saxon Incubator of Clinical Translation (SIKT, Leipzig, Germany). All rats had free access to water and were fed with a regular chow food (Sniff, Soest, Germany). Body weight was recorded for each group (WOKW mean 673 g, p < 0.05; LEW.1W mean 503 g, p < 0.05).

Experiments followed the international guidelines of animal care and the study protocols were approved by the Landesdirektion Leipzig, the local authority for animal care (T01/13 and T08/16). All rats were sacrificed via an isoflurane overdose at the end of the experiment.

### Phenotypic characterization

At an age of 5 months all animals were weighed, blood was collected and whole body composition (fat mass, lean mass and total body water) was determined in awake rats by using nuclear magnetic resonance technology with EchoMRI700™ instrument (Echo Medical Systems, Houston, TX, USA). Data were analyzed by the manufacturer’s software.

Serum insulin concentrations were measured by ELISA using Mercodia Rat ELISA (Uppsala, Sweden). Blood glucose values were determined from whole venous blood samples using an automated glucose monitor (FreeStyle mini, Abbott GmbH, Ludwigshafen, Germany). HbA1c levels were analysed by an automatic chemical analyzer in our Institute of Laboratory Medicine and Clinical Chemistry.

### Primary adipocyte cell cultures

WOKW and LEW.1W rats were anesthetized with 2.5% isoflurane inhalation. Subcutaneous and visceral adipose tissue depots were removed under sterile conditions, washed in PBS and incubated in a 5 ml of preadipocyte isolation buffer containing 123 mM NaCl, 5 mM KCl, 1.3 mM CaCl_2_, 5 mM Glucose, 100 mM HEPES, 4% BSA (bovine serum albumin fraction V) and 0.2% collagenase (type 1). Incubations were performed in a water bath with rotating flasks (120 rpm) at the temperature 37 °C for 40 min. After incubation the tissue remnants and cell aggregates were removed by filtration through a 100 µm nylon screen. The cells passing the filter were pelleted by centrifugation (5 min, 1500 rpm at RT) and then resuspended in an erythrocyte lysis buffer containing 0.154 M NH_4_Cl, 0.01 M KHCO_3_ and 0.1 mM EDTA for 10 min at RT. After incubation the preadipocyte pellet was resuspended and maintained in high-glucose Dulbecco’s modified Eagle’s Medium (DMEM) supplemented with 10% fetal bovine serum (FBS, Sigma) at 37 °C in a 5% CO_2_ atmosphere. Approximately 20,000 cells per well of a 24-well plate or per 30-mm culture dishes were obtained.

At day 2 of confluence, preadipocytes were induced to differentiate into adipocytes by using medium with 1 mM insulin, 0.4 mg/ml dexamethasone and 0.5 mM 3-isobutyl-1-methylxanthine changed every 48 h. After additional 7 days, about 85% of the cells had accumulated large-sized fat droplets as verified by phase contrast microscopy. For studies of autophagy dependent regulation of adiponectin, MCP-1, TNFα, RNF157 and cleaved caspase-3 expression, adipocytes were used at day 9 after the induction of differentiation.

### Inhibition of the PI3K pathway

Phosphorylation of phosphatidylinositol 3-kinase (PI3K) is required for autophagy activation, synthesis of autophagosomes and lipidation of cytosolic LC3-I into the active, autophagosome membrane-bound form, LC3-II. LY294002 is a specific inhibitor of PI3K phosphorylation [20].

**Fig. 1:**
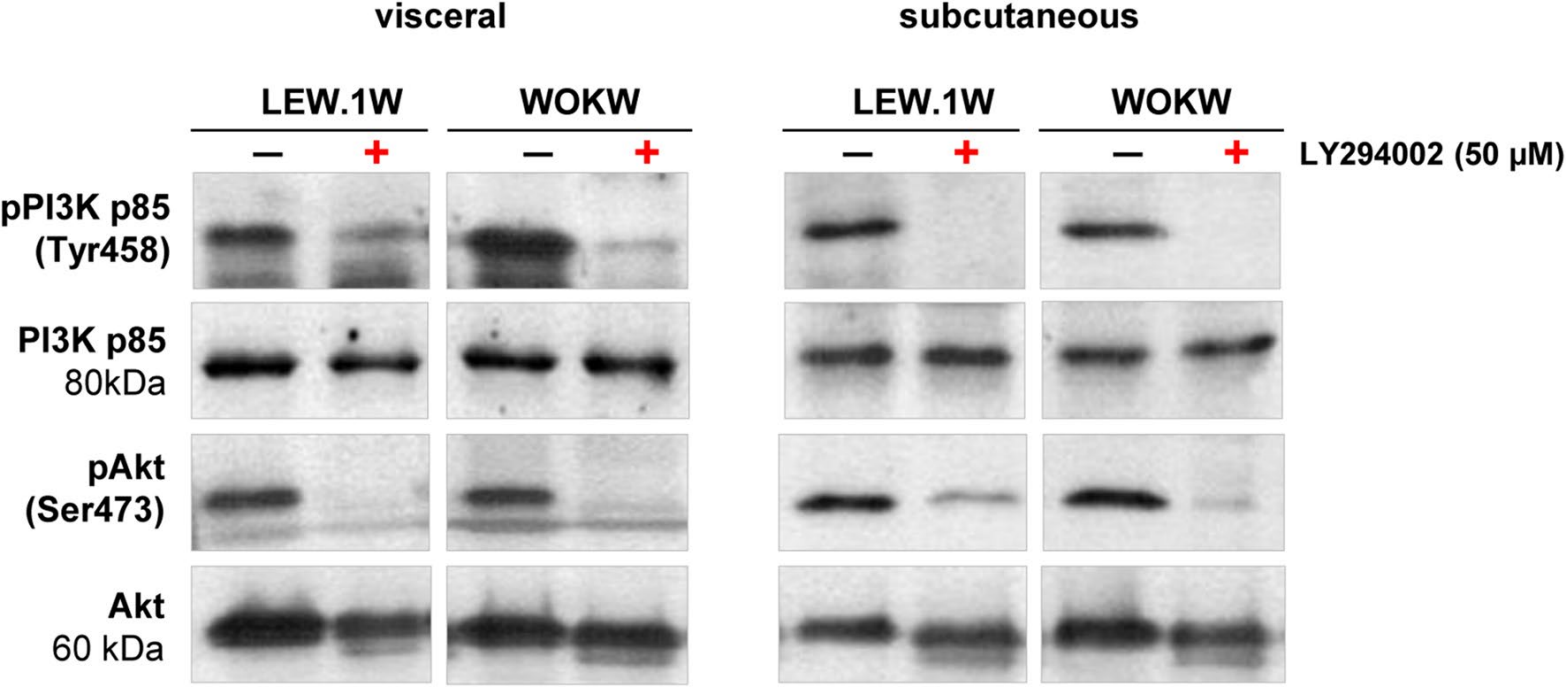
Inhibition of PI3K-dependent autophagy activation using an LY294002 inhibitor (50 µM) in cultivated adipocytes of WOKW and LEW.1W rats. Representative Western blots of PI3K p85, pPI3K p85, Akt and pAkt in cultured adipocytes, untreated (−) or treated (+) with LY294002 (50 µM). Note that inhibition of the PI3K signaling pathway for 45 min resulted in significant inhibition of phospho-PI3K and phospho-Akt expression in visceral and subcutaneous adipocytes of WOKW and LEW.1W rats

Dose-dependent inhibitory effect of LY294002 on PI3K phosphorylation was studied by treating adipocyte cells with increasing concentrations of LY294002 (20, 30 and 50 µM) in serum-free medium for 48 h. Completely inhibited phosphorylation was obtained at the concentration of 50 µM. To inhibit autophagy process, visceral and subcutaneous adipocytes were incubated in serum-free DMEM medium at 37 °C in the presence or the absence of LY294002 at a concentration of 50 µM for 48 h (Fig. 1a).

### Immunofluorescence

Visceral and subcutaneous adipocyte cultures of WOKW and LEW.1W rats (total number = 32; n = 4, per group) were fixed with 2% formaldehyde in 0.1 M PBS and 5% sucrose at 37 °C for 15 min. For detection of adiponectin and LC3 protein expression in adipocytes, double staining was performed using rabbit polyclonal anti-perilipin A (1 µg/ml; ab3526; Abcam, Cambridge, MA) and mouse monoclonal anti-adiponectin (2 µg/ml; ab22554; Abcam) or mouse anti-LC3 (2 μg/ml; clone 5F10; nanoTools, Munich, Germany) antibodies at 4 °C overnight. For detection of RNF157 (ring finger protein 157) protein expression in cultivated adipocytes a single staining with a rabbit polyclonal anti-RNF157 antibody (2 µg/ml; bs-9226R; Bioss, antibodies-online GmbH, Germany) was conducted. After rinsing with buffer, CY3-conjugated goat anti-mouse and/or FITC conjugated goat anti-rabbit antibodies (2 μg/ml; Dianova, Hamburg, Germany) were applied at room temperature (RT) for another 2 h. The coverslips were mounted with Glycerol (DAKO, Hamburg, Germany) containing DAPI 10 μg/ml (Serva, Heidelberg, Germany) for nuclear staining and DABCO 25 μg/ml (Sigma Aldrich) to prevent photobleaching. By replacement of the primary antibody with normal mouse IgG or PBS, respectively, no specific immunoreaction occurred.

All immunostaining images were taken using a Zeiss LSM 700 confocal microscope, and the ZEN 2011 software. The following laser settings were used: scan speed-9, pixel dwell 1.58 μs, scan time 11.62 s, number of scans-4, Bit depth-8 bit, DAPI track-laser (405 nm, pinhole-3.22 airy units, gain-766), FITC track-laser (488 nm, pinhole 1.90 airy units, gain-733) and CY3 track-laser (555 nm, pinhole 1.70 airy units, gain-633).

### Western blot

For preparing cell cultures lysates, cultured adipocytes (total number = 24; n = 6, per group) were washed in PBS and homogenized in lysis buffer containing 50 nM Tris, 150 nM NaCl, 1% NP-40, 0.5% Na-deoxycholate and 0.1% SDS. Samples were then incubated over 1 h in ice and centrifuged at 13,000×*g* for 15 min at 4 °C. Protein concentration was assessed with the BCA protein assay (Pierbo Science, Bonn, Germany). Proteins (20 μg per lane) were separated by electrophoresis on 10 or 15% SDS–polyacrylamide gels and transferred to nitrocellulose membranes by electroblotting. Nonspecific binding sites were blocked with 5% dry milk for 50 min. The blots were incubated with different antibodies at 4 °C overnight: mouse anti-LC3 (0.5 μg/ml; clone 2G6; nanoTools, Munich, Germany), mouse anti-TNF alpha (tumor necrosis factor alpha; 0.5 μg/ml; ab6671; Abcam, MA, USA), rabbit polyclonal anti-MCP1 (monocyte chemoattractant protein-1; 2 µg/ml; ab25124; Abcam), mouse monoclonal anti-adiponectin (2 µg/ml; ab22554; Abcam), rabbit polyclonal anti-RNF157 (1 µg/ml; bs-9226R; Bioss, antibodies-online GmbH, Germany), rabbit anti-cleaved caspase-3, Atg5 (D1G9), anti-mTOR, rabbit anti-Akt, mouse anti-pAkt, rabbit anti-PI3K, mouse anti-pPI3K (1 μg/ml; Cell Signaling Technology, Danvers, USA). Proteins were detected by incubating with HRP conjugated secondary antibodies (0.5 μg/ml; Dianova) at RT for 2 h and chemiluminescence kit (Amersham, Pharmacia, Freiburg, Germany). Integrated optical densities of the immunoreactive protein bands were measured with Gel Analyzer software (Media Cybernetics, Silver Spring, MD). Equal protein loading was verified using mouse anti-d-glyceraldehyde-3-phosphate dehydrogenase antibody (GAPDH; 0.2 μg/ml; Research Diagnostics, Flanders, The Netherlands). The extract of Jurkat cells with cytochrome c-induced apoptosis (10 μg; Cell Signaling Technology) was used as positive control for the detection of cleaved caspase-3.

### RT-PCR analyses

For mRNA analyses, subcutaneous and visceral AT depots of WOKW and LEW.1W rats (n = 4) were removed under 2.5% isoflurane anesthesia, transferred on ice, rinsed in PBS and immediately frozen in liquid nitrogen. mRNA expression of rat marker genes of *Rnf157* and *Mcp*-*1* was measured by quantitative real-time RT-PCR in a fluorescent temperature cycler using the TaqMan assay, and fluorescence was detected on an ABI-PRISM 7000 sequence detector (Applied Biosystems, Darmstadt, Germany). Total RNA was isolated using TRIzol (Life Technologies, Grand Island, NY). 1 μg RNA was reverse transcribed with standard reagents (Life Technologies). From each RT-PCR consisting of an initial denaturation at 95 °C for 10 min, followed by 40 PCR cycles, each cycle consisting of 95 °C for 15 s, 60 °C for 1 min, and 72 °C for 1 min. 2 μl was amplified in a 20-μl PCR using the Brilliant SYBR Green PCR mix from Thermo Fisher Scientific (Waltham, MA, USA). The following primers were used: rat *Rnf157*, 5′-AACAGCCAAGGGCTCAAACT-3′ (sense) and 5′-TCTGACTCACTGCAAGAGCG-3′ (antisense); rat *Mcp*-*1*, 5′-TAGCATCCACGTGCTGTCTC-3′ (sense) and 5′-CAGCCGACTCATTGGGATCA-3′ (antisense) (Biomers, Ulm, Germany). Rat *Rnf157* and *Mcp*-*1* mRNA expression was calculated relative to the mRNA expression of 18S rRNA, determined by a premixed assay on demand for human 18S rRNA (Hs99999901_s1; Life Technologies, CA). Amplification of specific transcripts was confirmed by melting curve profiles (cooling the sample to 60 °C and heating slowly to 95 °C with measurement of fluorescence) at the end of each PCR. Specificity of PCR was verified by subjecting the amplification products to agarose gel electrophoresis, which verified the presence of a single band of appropriate molecular weight for each amplification product.

### Statistical analyses

Data are presented as mean ± SEM (Figs. 1, 3, 4, 5, 7). Differences between the groups were validated by one-way-ANOVA and the Newman–Keuls test using SigmaStat (Jandel Scientific, San Rafael, CA). A value of p < 0.05 was considered statistically significant.

## Results

### Phenotypic characterization of WOKW rats

The phenotype of the 5-months-old WOKW (n = 24) and LEW.1W control rats (n = 24) is summarized in Table 1. WOKW rats have a significantly higher body weight as well as fasting serum insulin concentrations compared to healthy LEW.1W rats. In contrast, HbA1c levels and fasted blood glucose did not differ between both strains. The adiposity index was significantly increased in WOKW compared to LEW.1W rats (Table 1).

**Table 1:** Characteristics of LEW.1W and WOKW rats at an age of 5 months (mean ± SEM)

Traits	LEW.1W (n = 24)	p value	WOKW (n = 24)
Body weight (g)	503.3 ± 12.1	< 0.001	673.4 ± 21
Fat mass (g)	58.6 ± 18	< 0.01	181.6 ± 27
% body fat	11.5 ± 3.3	< 0.001	31.1 ± 8.6
Blood glucose (mmol/l)	5.8 ± 0.5	NS	6.5 ± 0.9
HbA1c (%)	3.7 ± 0.3	NS	3.8 ± 0.2
Serum insulin concentration (ng/ml)	1.3 ± 0.6	<0.001	5.1 ± 0.6

### Inhibition of autophagy in visceral and subcutaneous adipocyte cell cultures of WOKW and LEW.1W rats

We have previously shown the strikingly up-regulated autophagy in adipose depots of obese WOKW rats with metabolic syndrome in correlation with inhibition of caspase-3 apoptotic signal pathway [15].

Here, we extent these findings by pharmacologically inhibiting autophagy in cultured adipocytes of obese WOKW rats with MetS and in lean LEW.1W control healthy rats. Mature visceral and subcutaneous adipocytes from both strains have been inhibited with an autophagy inhibitor—LY294002 at the concentration of 50 µM for 48 h.

As shown in Fig. 1, phosphorylation of key autophagy signaling pathway—PI3K p85 and Akt protein in visceral and subcutaneous adipocytes of WOKW and LEW.1W rats was inhibited with LY294002 inhibitor after 45 min of treatment. Next, we assessed the protein level of the autophagy related genes—Atg5/12, LC3 and mTOR in adipocyte cell cultures after inhibition.

**Fig. 2:**
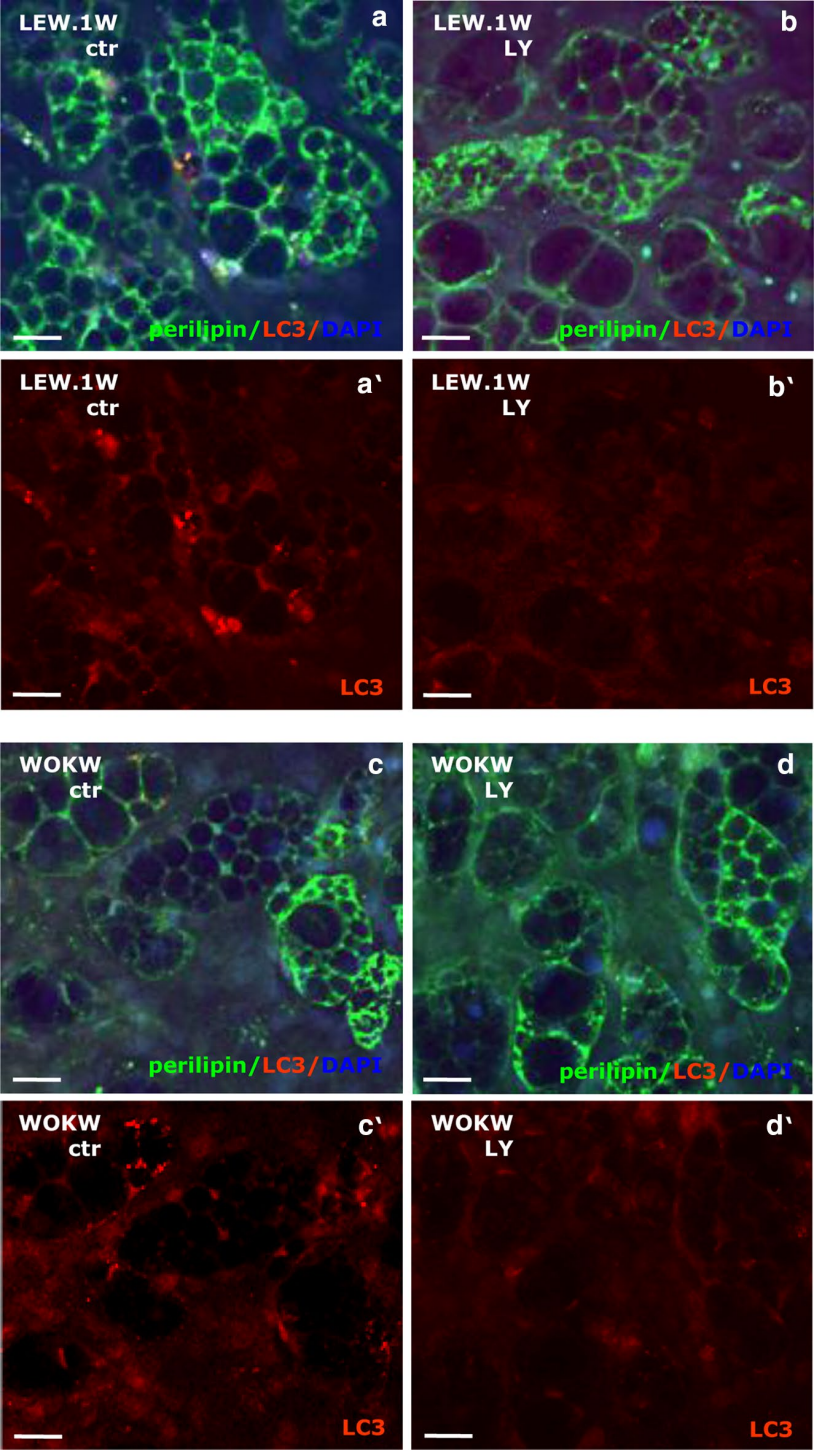
Effect of autophagy inhibition on LC3-autophagy marker expression in visceral adipocytes of WOKW and LEW.1W rats. Perilipin A (green, limited to adipocytes) with the microtubule-associated protein light chain 3 (LC3, red) were detected in visceral adipocytes (higher autophagic activity) of WOKW and LEW.1W rats using double immunofluorescence staining (**a**–**a′**, **d**–**d′**). After inhibition of PI3K signaling pathway the immunoreactivity of LC3 positive vesicles was not detectable (**b′**, **d′**). Nuclei are counterstained with DAPI (blue)

Visceral adipocyte cell cultures of WOKW and LEW.1W rats display a significant decrease of LC3 immunoreactivity compared to the non-inhibited control cultures after inhibition with LY294002 for 48 h (Fig. 2a–d).

**Fig. 3:**
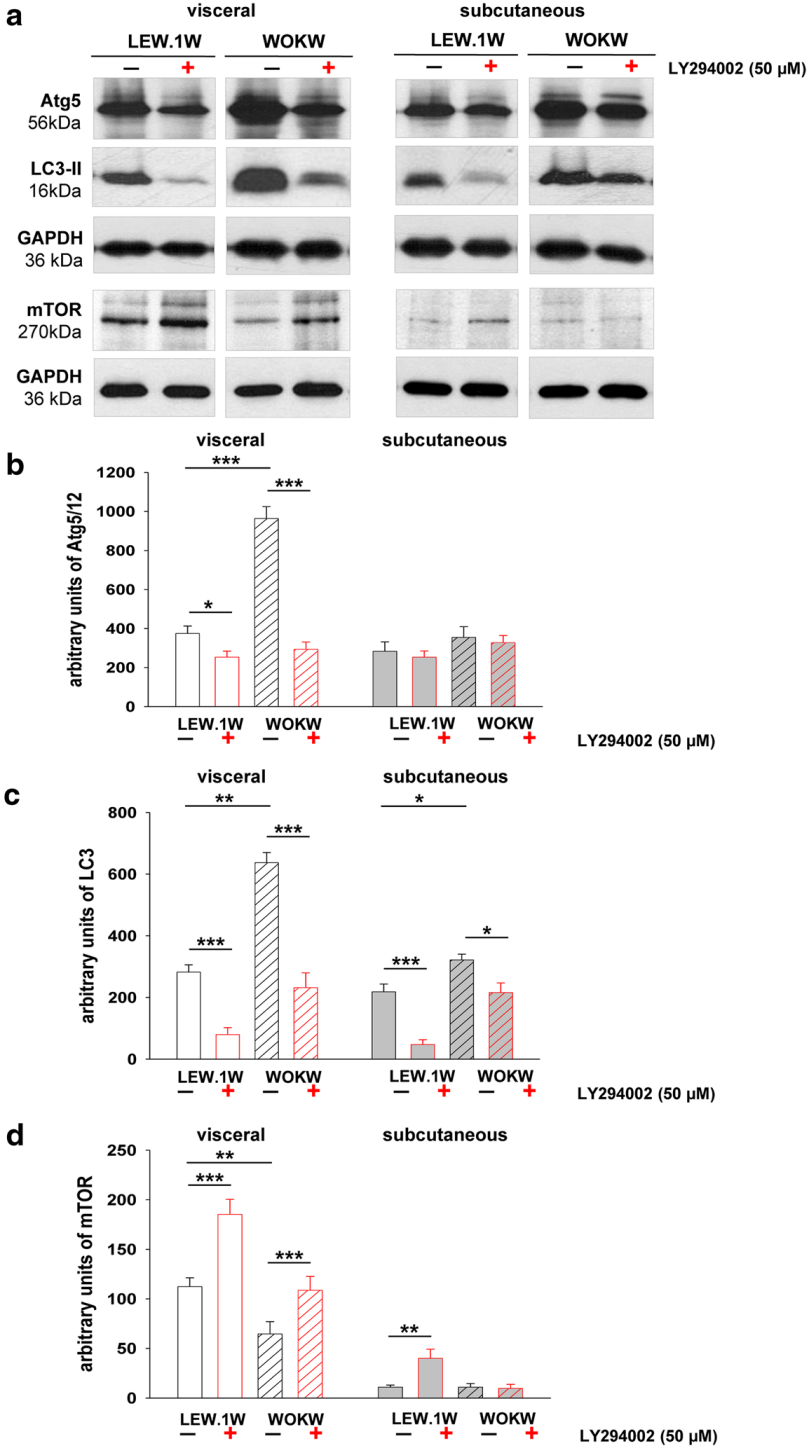
Detection of the autophagy markers: Atg5-12, LC3-II and mTOR in visceral and subcutaneous adipocytes of WOKW and LEW.1W rats, untreated (−) or treated (+) with LY294002 (50 µM). Representative Western blots (**a**) and corresponding densitometrical analyses (**b**, **c**, **d**) of autophagy-related gene 5-12 (Atg5-12), microtubule-associated protein light chain 3 (LC3) and mammalian target of rapamycin (mTOR). **a**–**c** Increased expression of Atg5-12 and LC3-II (membrane bound) protein in visceral and subcutaneous adipocytes of WOKW rats compared to the LEW.1W control animals. Note the significantly decreased Atg5-12 and LC3 expression after PI3K inhibition with LY294002 (48 h) in visceral adipocytes of both strains. **a**, **d** Downregulation of mTOR was detected predominantly in the visceral adipocytes of WOKW rats. The treatment with LY294002 (48 h) resulted in an increase in mTOR expression in visceral adipocytes of both strains. Data from n = 6 are presented as mean ± SEM. *p ≤ 0.05, **p ≤ 0.01, ***p ≤ 0.001, according to the one-way analysis of variance together with the Newman–Keuls test. GAPDH was used as normalization control

Western blot analyses confirmed an inhibitory effect of LY294002. Expression of Atg5/12 protein was decreased about threefold in visceral adipocytes of WOKW rats and 1.3-fold in LEW.1W rats (Fig. 3a, b). LC3-II (membrane associated, 16 kDa form of LC3) displayed a 2.6-fold down-regulation in visceral adipocytes of WOKW rats and a 3.7-fold decrease in LEW.1W rats. In subcutaneous adipocytes, a 1.6-fold down-regulation of LC3 protein expression was found in WOKW rats and 4.5-fold decrease in LEW.1W control rats (Fig. 3a, c). Expression of mTOR was increased about 1.6-fold in visceral adipocytes of both strains. Contrary, in subcutaneous adipocyte cell cultures only LEW.1W rats show higher expression of mTOR after autophagy inhibition (Fig. 3a, d).

### Autophagy dependent regulation of adiponectin, MCP-1 and TNFα protein expression in cultured adipocytes of WOKW and LEW.1W rats

In the obese state, the adipocytes increase secretion of pro-inflammatory chemokines and cytokines, such as MCP-1, TNFα or interleukins (ILs) which are involved in insulin resistance [21–23]. Moreover, the expression of the insulin-sensitizing adipokine—adiponectin is strikingly down-regulated in obesity [24]. AT protein level of adiponectin correlates with up-regulated autophagy and was lower in cultured adipocytes of obese WOKW rats as compared to LEW.1W control rats.

**Fig. 4:**
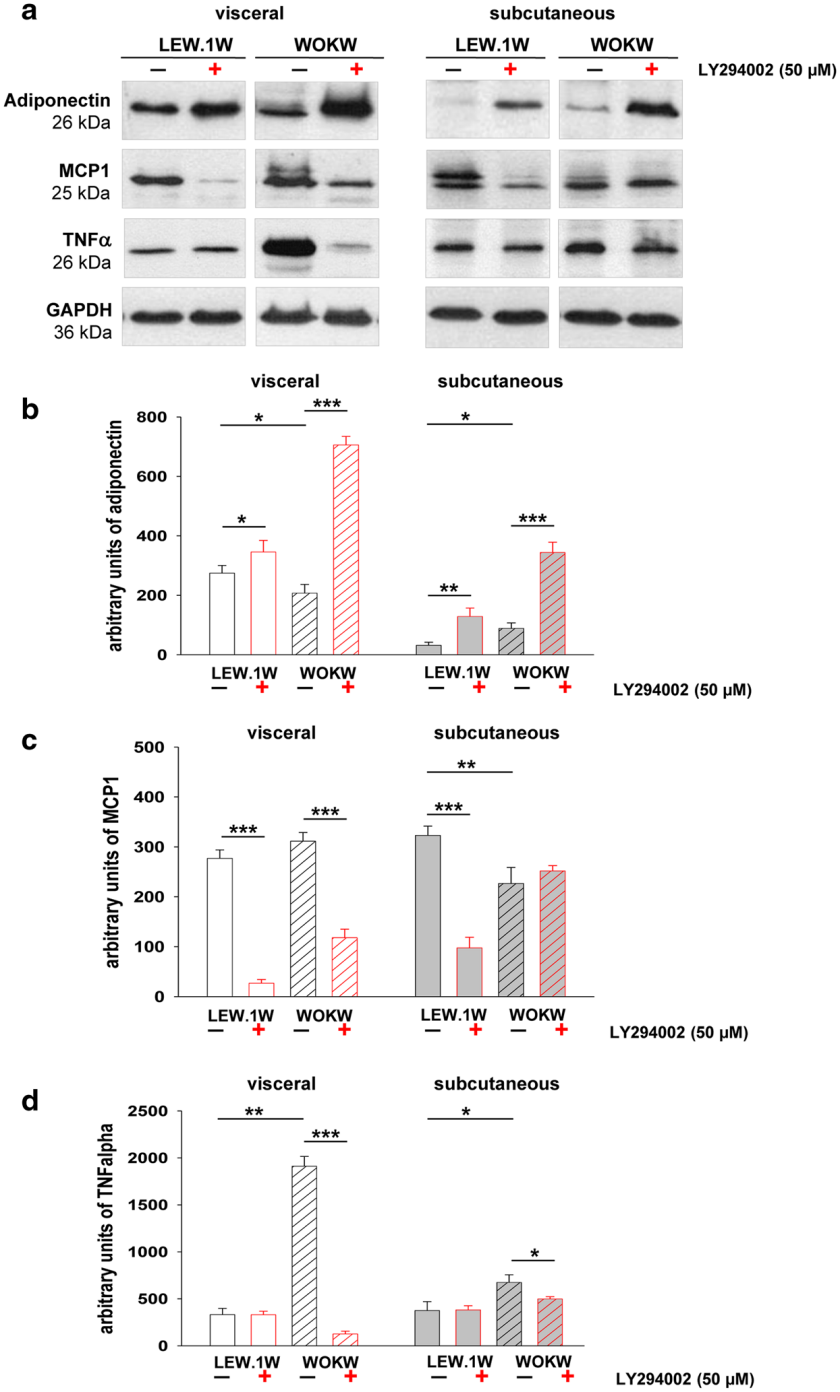
Expression of proinflammatory markers: MCP1 and TNFα and protective adiponectin in cultivated adipocytes of WOKW and LEW.1W rats. Representative Western blots (**a**) and corresponding densitometrical analyses of adiponectin, MCP1 and TNFα (**b**–**d**). **a**, **b** Western blot analysis revealed the decreased expression of adiponectin in visceral adipocytes of obese WOKW rats with MetS as compared to lean LEW.1W healthy rats. The adiponectin expression in subcutaneous adipocytes of both strains was slightly detected. The inhibition of PI3K-dependent autophagy pathway resulted in significantly upregulation of adiponectin levels, pronounced in adipocytes of WOKW rats. **a**, **c**, **d** Increased expression of MCP1 and TNFα protein was predominantly detected in visceral adipocytes of WOKW rats as compared to LEW.1W rats. These proteins could be significantly downregulated through autophagy inhibition (48 h) in both strains. Data from n = 6 are presented as mean ± SEM. *p ≤ 0.05, **p ≤ 0.01, ***p ≤ 0.001, according to the one-way analysis of variance together with the Newman–Keuls test. GAPDH was used as normalization control

After autophagy inhibition (48 h), adiponectin showed an about 3.5- and 4-fold (WOKW rats) and 1.2- and 3.6-fold (LEW.1W rats) up-regulation in visceral and subcutaneous adipocyte cell cultures (Fig. 4a, b).

Moreover, we confirm the up-regulated mRNA (Additional file 1: Figure S1) and protein expression of MCP-1 in adipose depots of WOKW rats in comparison to LEW.1W control rats mainly in visceral adipocytes (Fig. 4a, c). MCP-1 protein expression was decreased in visceral adipocytes of both strains after LY294002 treatment. In contrast, MCP-1 protein in subcutaneous adipocytes is down-regulated in LEW.1W rats only (Fig. 4a, c).

Additionally, we have found a fourfold increase in TNFα protein expression in visceral adipocyte cell culture of WOKW rats, which could be significantly attenuated after autophagy inhibition. The expression of TNFα in subcutaneous adipocytes has been detected at comparable levels between all experimental groups (Fig. 4a, d).

**Fig. 5:**
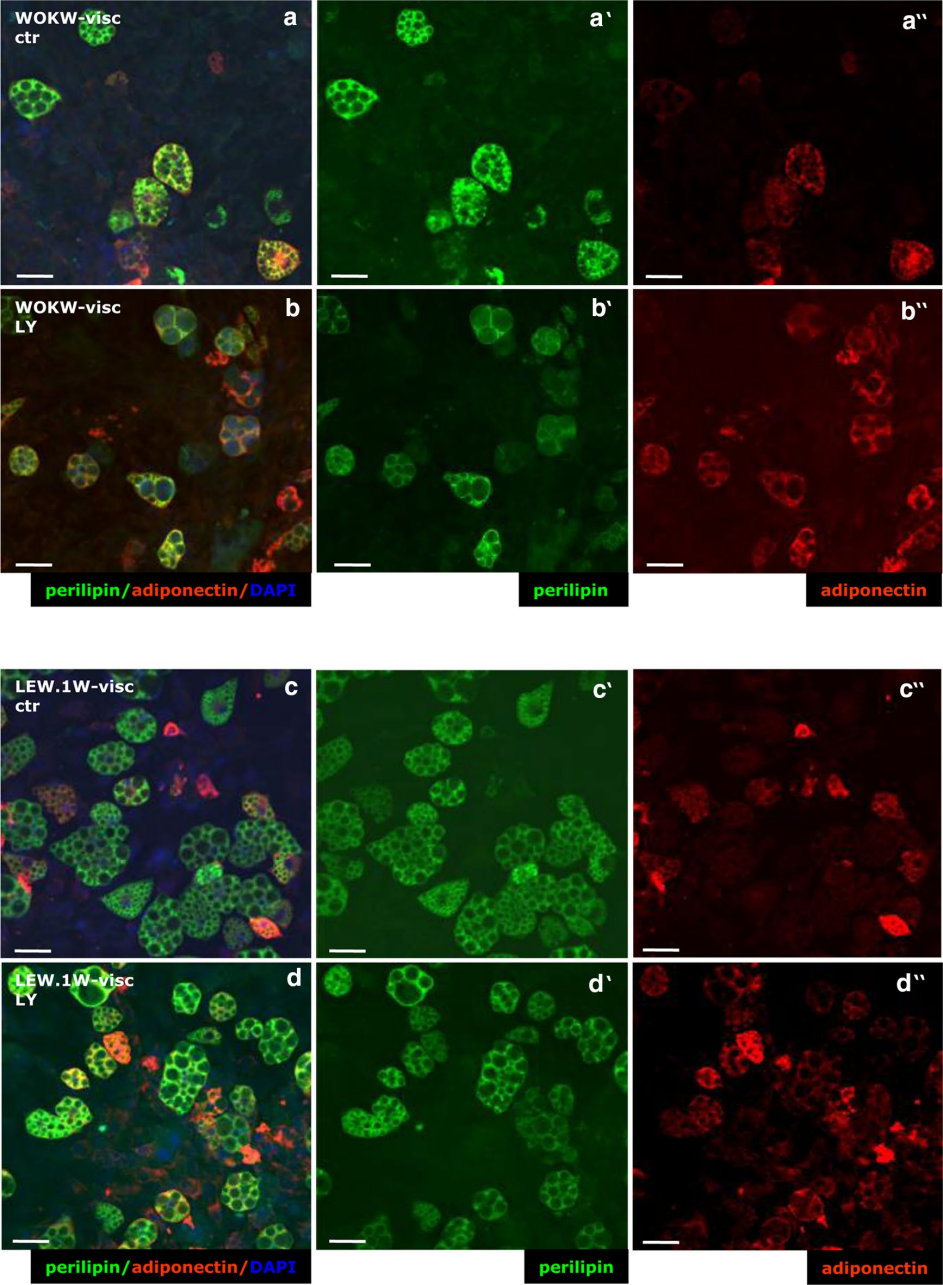
Detection of adiponectin immunoreactivity after autophagy inhibition in visceral adipocytes of WOKW and LEW.1W rats. **a**–**d″** Adionectin (red) with perilipin A (green) were detected in visceral adipocytes (higher autophagic activity) of WOKW and LEW.1W rats using double immunofluorescence staining Note the adiponectin immunoreactivity was lower in visceral adipocytes of WOKW rats than in LEW.1W rats (**a**–**a″**, **c**–**c″**). In accordance with Western blot data, the inhibition of PI3K signaling pathway and autophagic activity resulted in strikingly increase of adiponectin immunoreactivity (positive adipocytes) of both strains, with prevalence to WOKW rats (**b**–**b″**, **d**–**d″**). Nuclei are counterstained with DAPI (blue)

As shown in Fig. 5a–d immunohistofluorescence analysis of adiponectin distribution in visceral adipocyte cell cultures demonstrating an increase of adiponectin immunoreactivity after LY294002 treatment. Immunofluorescence of adiponectin in subcutaneous adipocytes was rarely detectable.

### Regulation of RNF157 in adipocyte cell cultures in obese WOKW rats

**Fig. 6:**
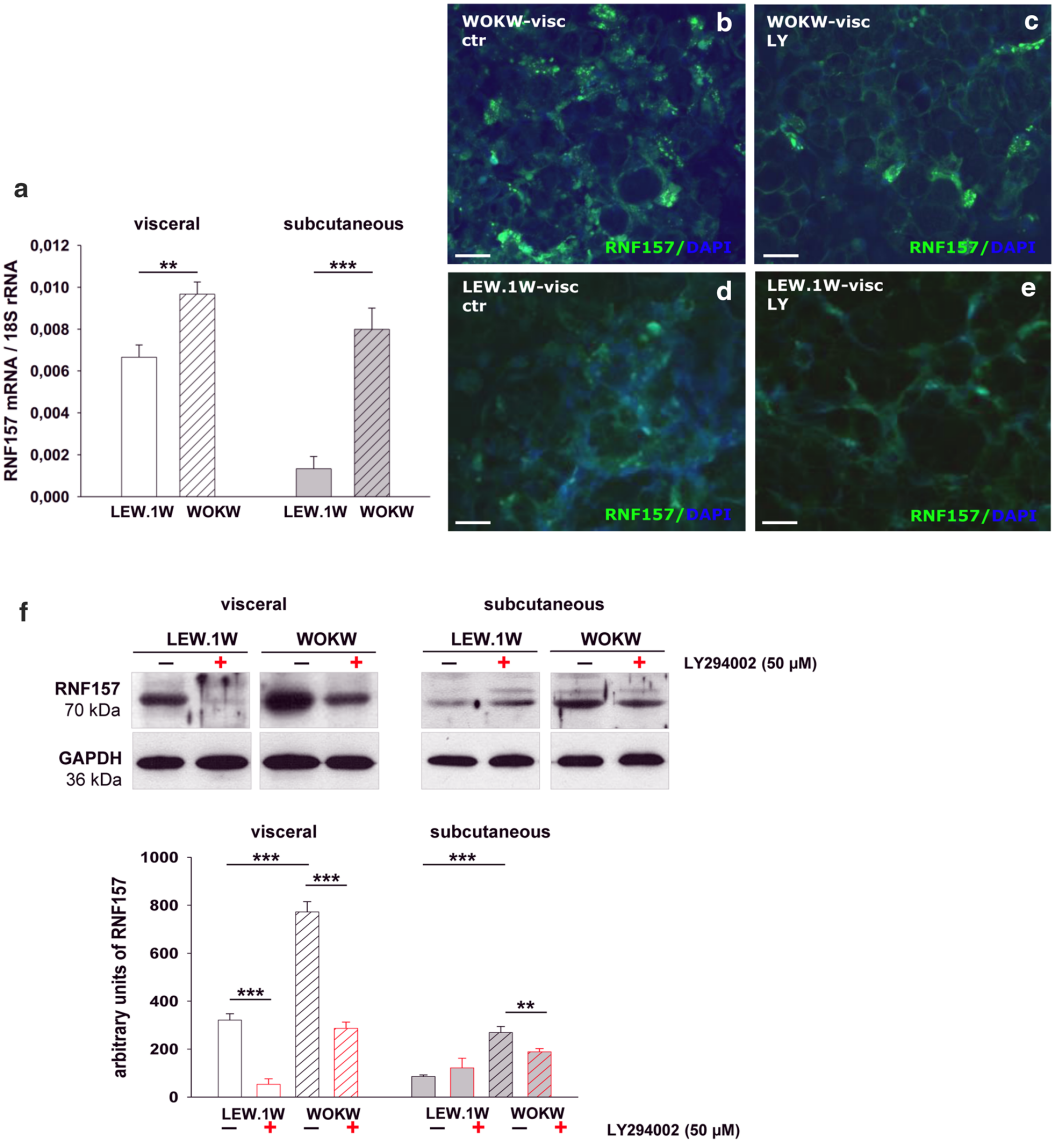
Expression of autophagy related RNF157 protein in visceral and subcutaneous fat depots and cell cultures of WOKW and LEW.1W rats. **a** RNF157 mRNA expression was increased in the visceral and subcutaneous fat depots of obese WOKW rats with MetS as compared to lean LEW.1W healthy control rats. The higher mRNA expression of RNF157 was noted in visceral AT vs. subcutaneous in both strains. Data from four individuals per group are presented as mean ± SEM. *p ≤ 0.05, **p ≤ 0.01, ***p ≤ 0.001, according to the one-way analysis of variance together with the Newman–Keuls test. **b**–**e** Detection of RNF157 protein expression with immunofluorescence staining (green) in visceral adipocyte cell cultures of WOKW and LEW.1W. The higher immunoreactivity of RNF157 was found in adipocyte cultures of WOKW rats vs. LEW.1W rats. The markedly decrease of RNF157 expression was noted after autophagy inhibition with LY294002 (48 h). Nuclei are counterstained with DAPI (blue). **f** Representative Western blots and corresponding densitometrical analyses of RNF157. Western blot analysis revealed the significantly increased expression of RNF157 in visceral adipocytes of obese WOKW rats with MetS as compared to lean LEW.1W healthy rats. The RNF157 expression in subcutaneous adipocytes of both strains was slightly detected. The inhibition of PI3K-dependent autophagy pathway resulted in significantly downregulation of RNF157 levels, pronounced in adipocytes of WOKW rats. Data from n = 6 are presented as mean ± SEM. *p ≤ 0.05, **p ≤ 0.01, ***p ≤ 0.001, according to the one-way analysis of variance together with the Newman–Keuls test. GAPDH was used as normalization control

Unexpectedly, a strikingly high mRNA expression of new candidate gene—*Rnf157*, a neuroprotective protein [25], has been detected in visceral and subcutaneous depots of WOKW rats and in visceral fat of LEW.1W rats (Fig. 6a–f).

Interestingly, we found a 5- and 2.6-fold decrease of RNF157 protein expression after autophagy inhibition in visceral adipocyte cell cultures of WOKW and LEW.1W rats, respectively. Subcutaneous adipocytes of WOKW rats exhibit a 1.4-fold decrease in RNF157 expression after treatment. No changes in RNF157 expression were observed in subcutaneous adipocyte cultures of LEW.1W rats after autophagy inhibition (Fig. 6f). Inhibition of autophagy resulted in decrease of RNF157 immunoreactivity in visceral adipocytes of both strains (Fig. 6b–e).

### Up-regulation of cleaved caspase-3 signal pathway after inhibition of autophagic activity

**Fig. 7:**
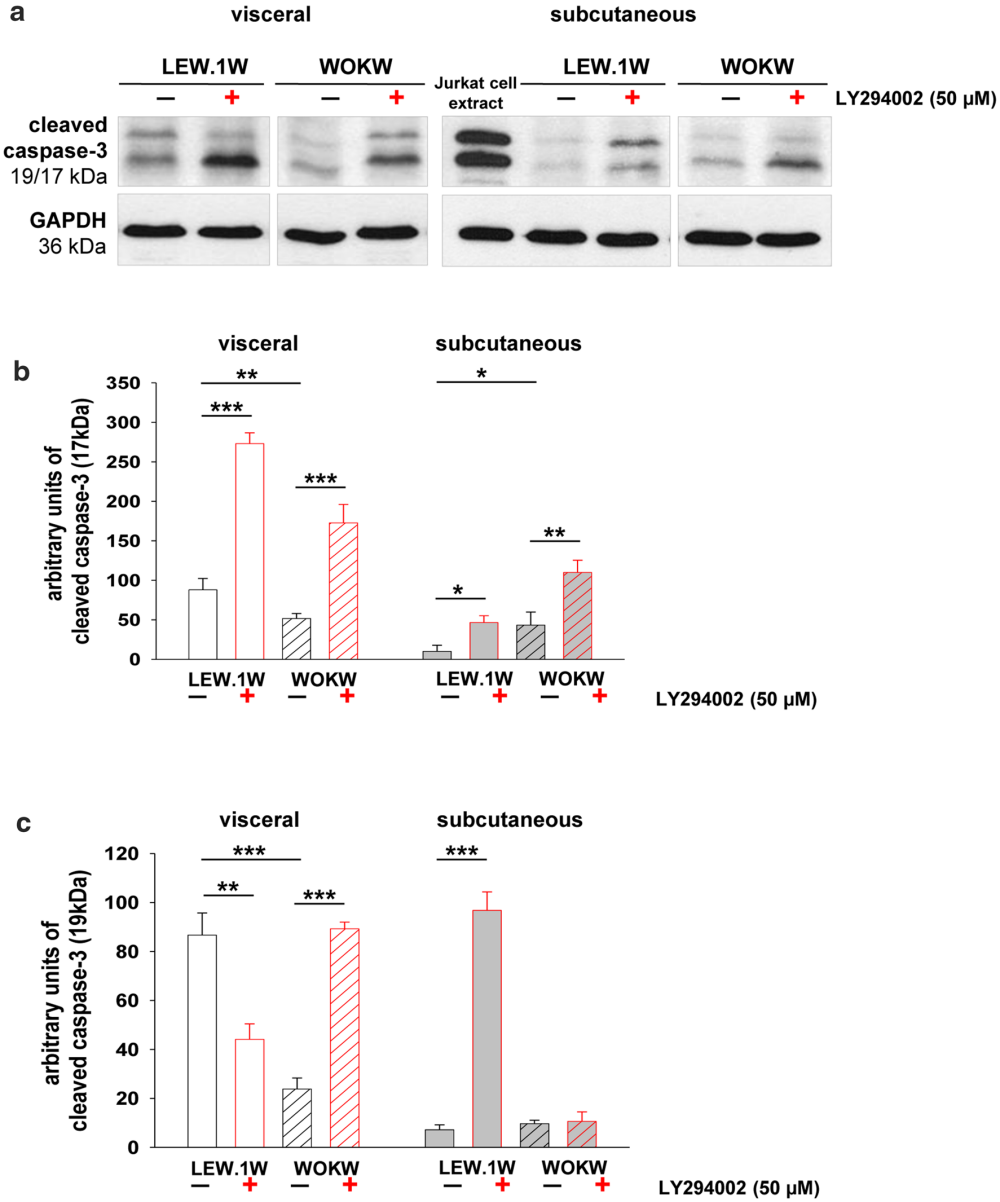
Regulation of the apoptosis marker cleaved caspase-3 under PI3K inhibition. Representative Western blots (**a**) and corresponding densitometrical analyses of cleaved caspase-3 (**b**–**c**). The cleaved caspase-3 expression in adipocyte cell cultures of both strains was slightly detected by Western blot. The inhibition with LY294002 (48 h) caused significantly increase of cleaved caspase-3 expression (17 kDa, mature form of enzyme) in visceral and subcutaneous adipocytes. An extract of Jurkat cells with cytochrome c-induced apoptosis was used in Western blot as the positive control for the occurrence of cleaved caspase-3 (**a**). Data from n = 6 are presented as mean ± SEM. *p ≤ 0.05, **p ≤ 0.01, ***p ≤ 0.001, according to the one-way analysis of variance together with the Newman–Keuls test. GAPDH was used as normalization control

Since autophagy promotes survival or once exhausted could activate apoptosis [26], cleaved caspase-3, an apoptosis marker, has been assessed in cultivated adipocytes in response to autophagy inhibition. Western blot analysis demonstrate significantly increased cleaved caspase-3 (17 kDa subunit) protein expression after autophagy inhibition in both adipocyte cell cultures of WOKW and LEW.1W rats predominantly in visceral adipocytes (Fig. 7a–c).

## Discussion

Obesity and metabolic syndrome (MetS) have been linked with enhanced autophagic activity in AT [7]. Increased expression of autophagy genes correlates with visceral fat distribution, adipocyte hypertrophy, secretion of pro-inflammatory adipokine and contributes to adipose tissue (AT) inflammation [7, 27, 28]. There is a complex bidirectional relationship between autophagic activity and inflammation. Autophagy pathway induces or suppresses inflammatory responses, whereas inflammatory signals regulate the autophagic flux [12, 29, 30]. Inflammatory activation in obesity occurs principally in metabolic tissues including AT, liver and pancreas [5]. Expansion of AT with predominant adipocyte hypertrophy triggers the secretion of pro-inflammatory chemokines and causes obesity-induced inflammation. Among them, MCP-1, TNFα, interleukin IL-1 and IL-6 have been reported to promote obesity related-insulin resistance [28].

Obesity-related AT inflammation and insulin resistance result in a decrease of anti-inflammatory adipokines, such as adiponectin [28, 31, 32]. Slutsky, Rudich et al. have reported the association between decreased adiponectin and elevated autophagy in omental AT of patients with obesity [33]. However, obesity-related inflammation with up-regulated autophagy has been assumed as a destructive factor, the impact of autophagy as a protective mechanism in obesity is carefully discussed. Codogno and Meijer argued that initially obesity-induced insulin resistance functions as an adaptive mechanism to increase autophagy in order to protect cells against death [34]. Activated autophagy characterized by an increased expression of autophagy genes ATG5, LC3A, and LC3B as well as elevated autophagic flux in omental and subcutaneous adipose tissue has been reported in non-diabetic obese individuals more pronounced as in type 2 diabetes (T2D) patients [7, 9, 35]. Our previous data confirmed that autophagy contributes to AT inflammation in obesity and its related metabolic comorbidities [7, 9, 15, 16]. Increased autophagy in obese and T2D patients compared to lean individuals is reflected by a higher infiltration of Iba-1 positive macrophages and up-regulation of TNFα and IL-6 expression in visceral AT. Interestingly, increased autophagy and inflammation in visceral AT of obese patients without T2D corresponded with inactivation of cleaved-caspase 3 dependent apoptosis pathway [9]. Autophagy occurring parallel to inflammation is assumed as a protective factor, since it does not invoke apoptosis. Correspondingly, we found a significant increased autophagy with macrophage infiltration in the sciatic nerves and visceral AT of adult, obese WOKW rats with MetS [15, 16]. Notably, WOKW rats do not develop any age-related MetS complications such as diabetes or neuropathy. Therefore, we proposed that up-regulated autophagy exerts a protective role against development of diabetes and peripheral neurodegeneration in the WOKW rat model.

*The present study* highlights the impact of autophagy on adipocyte function in cultured adipocytes of obese WOKW rats with MetS. We demonstrate that inhibition of autophagy leads to enhanced expression of protective and inflammatory factors and induces the expression of cleaved caspase-3 apoptotic marker in adipocytes of WOKW rats as compared to the lean, healthy control LEW.1W rats.

In accordance with our previous findings, significantly up-regulated autophagy has been detected in cultured adipocytes of obese WOKW rats as compared to LEW.1W. We found a markedly higher expression of autophagic molecular markers, including Atg5-Atg12 complex, lipidated/cleaved form of LC3 (LC3-II), and down-regulation of mTOR protein, respectively.

Up-regulated autophagy in visceral adipocytes of WOKW rats vs. LEW.1W corresponds with an increase of RNF157 protein expression. RNF157 could be co-regulated by autophagy mechanisms in adipose tissue or induce autophagy. Significantly higher mRNA and protein levels of RNF157 (about threefold) correlate with visceral AT mass, obesity and MetS parameters (WOKW vs. LEW.1W). Importantly, an inhibition of autophagic activity reduced the expression of RNF157 protein in AT of both strains. RNF157 is predominantly expressed in the brain, whereas non-neuronal tissues show little expression. This protein belongs to E3 ubiquitin RING ligases and is implicated in regulation of neuronal survival and morphology in cultured neurons [25]. In addition, a downstream component and substrate of RNF157—the adaptor protein Fe65 was found to trigger neuronal apoptosis. These unexpected observations of AT specific RNF157 expression and its correlation with activated autophagy require further mechanistic studies.

Among adipokines, adiponectin is an important modulator of glucose metabolism involved in insulin sensitivity and secretion [27]. Moreover, based on its anti-inflammatory properties, adiponectin has been classified as a protective adipokine [36]. Recently, up-regulated autophagy has been show to be implicated in impaired adiponectin secretion in omental AT of patients with obesity [33]. We have previously reported a tenfold reduced *adiponectin* mRNA in AT of obese WOKW rats as compared to healthy controls [19]. The present study revealed that autophagy significantly contributes to the regulation of adiponectin secretion in visceral and subcutaneous AT. Notably, lower adiponectin levels with up-regulated autophagy in cultured adipocytes (WOKW vs. LEW.1W) have been increased about fourfold after autophagy inhibition in adipocytes of WOKW rats. Whether the up-regulation of autophagy genes with attenuated expression of adiponectin in AT of WOKW rats mediates anti-metabolic or protective actions is questionable. The WOKW rats develop complete metabolic syndrome, but are resistant to MetS complications.

In order to investigate autophagy dependent regulation of pro-inflammatory cytokines, we focused particularly on MCP-1 and TNF-α mRNA and protein expression in AT and cultivated adipocytes of WOKW rats with MetS and LEW.1W control healthy rats. MCP-1, a member of the chemokine family, is a crucial factor implicated in chronic inflammation of visceral AT. An increased level of MCP-1 in obesity links to accumulation of M1-proinflammatory macrophages in visceral AT [37]. MCP-1 plays a crucial role in the recruitment of monocytes and T lymphocytes into tissues [38]. The resident stromal cells, monocytes, endothelial cells, preadipocytes and adipocytes have been determined to MCP-1 expression. More recently, adipocyte progenitor cells have been identified as the initial cellular source of MCP-1 [37]. A potentially direct action of MCP-1 on autophagy is unknown. In the present study, we confirmed that MCP-1 expression in visceral adipocytes is directly regulated through autophagic activity. The *MCP*-*1* mRNA levels were significantly higher in visceral and subcutaneous AT of WOKW rats as compared with LEW.1W rats. Correspondingly, higher MCP-1 protein expression was detected in cultured visceral adipocytes of WOKW rats vs. LEW.1W rats. The inhibition of autophagy resulted in significantly decrease of MCP-1 expression in visceral adipocytes of both strains. Differences between MCP-1 expression in AT (mRNA level) and in cultivated adipocytes (protein level) in WOKW rats vs. LEW.1W rats can be explained by the rare occurrence of macrophages or other components of stromal-vascular fraction in cell cultures as a source of MCP-1.

MCP-1, in concert with other pro-inflammatory cytokines such as TNF-α or IL-6 overexpressed by adipocytes in obesity and MetS, leads to insulin sensitivity. In the visceral adipocytes of WOKW rats, up-regulated autophagy corresponds to higher levels of TNFα, whereas the inhibition of autophagic activity significantly decreased TNFα expression. Perhaps, the autophagic process plays a regulatory function of cytokine dependent insulin sensitivity in visceral AT. The activation of autophagy pathways during inflammation has been reported to regulate pathogen clearance, antigen presentation, lymphocyte development and the production of proinflammatory cytokines [39]. The mechanistic relationship between autophagy and pro-inflammatory cytokine activation is dependent on the priority of occurring process, since one of these could precede the second.

In this study we demonstrate that an inhibition of autophagic activity significantly changed the expression of protective but also inflammatory factors and parallel markedly up-regulated the expression of cleaved caspase-3 in cultured adipocytes. However, the occurred changes were more evident in visceral adipocytes of obese WOKW rats than in LEW.1W healthy control rats. These results are compatible with our previous observation that increased autophagic activity protect from caspase-3 mediated apoptosis in AT of obese human and rats [9, 15]. The biological consequences of manipulation of autophagy process are dependent on cell type and metabolic context and can exert opposing effects. It has been assumed that “basal autophagy” as an adipocyte survival mechanism is essential for completion of adipogenesis. Defects in autophagy or its inhibition cause the higher level of apoptosis in AT and cultivated adipocytes (our observation). Notably, the autophagy related protein (Atg)-5 knockout mice die within the first day after birth. Atg-7 knockout mice are insulin-sensitive and resistant to the development of obesity [40]. Contrary, up-regulated autophagy is implicated in the obesity state and obesity-related inflammation. Therefore, autophagy represents distinctly regulated mechanism, which may protect against or develop obesity-associated AT dysfunction.

## Conclusion

Based on our previously observations about up-regulated autophagy in visceral AT of obese patients and WOKW rats with MetS in correlation with inflammation but not with apoptosis and T2D, we postulated that autophagy as a key protective rather as destructive factor. Here, we show the consequences of preventing autophagic activity in adipocytes, predominantly in visceral AT of WOKW rats as markedly changes of pro-inflammatory and protective adipokines parallel with activated apoptosis. This fact highlights the autophagy process for future research of diabetes prevention in obesity.

## Additional file

**Additional file 1: Figure S1.** Expression of *Mcp*-*1* mRNA in visceral and subcutaneous fat depots of WOKW and LEW.1W rats. *Mcp*-*1* mRNA expression was increased in the visceral and subcutaneous fat depots of obese WOKW rats with MetS as compared to lean LEW.1W healthy control rats. Data from four individuals per group are presented as mean ± SEM. *p ≤ 0.05, **p ≤ 0.01, ***p ≤ 0.001, according to the one-way analysis of variance together with the Newman–Keuls test.

## Abbreviations

Akt: protein kinase
pAkt: phospho-protein kinase
AT: adipose tissue
Atg5/12: autophagy related protein 5–12
CY3: cyanine 3
FITC: fluorescein isothiocyanate
GAPDH: d-glyceraldehyde-3-phosphate dehydrogenase
HbA1c: hemoglobin-A1c glycated hemoglobin
LC3: microtubule associated protein light chain 3
LEW.1W: Lewis 1W
LY294002: PI3K inhibitor
MCP1: monocyte chemoattractant protein-1
MetS: metabolic syndrome
mTOR: mammalian target of rapamycin
PI3K: phosphoinositide 3 kinase
pPI3K: phospho-phosphoinositide 3 kinase
RNF157: ring finger protein 157
T2D: type 2 diabetes
TNFα: tumor necrosis factor alpha
WOKW: Wistar Ottawa Karlsburg W
